# Study of Radioprotective Effect of Green Tea against Gamma Irradiation Using Micronucleus Assay on Binucleated Human Lymphocytes

**Published:** 2012

**Authors:** Hafezeh Davari, Farhang Haddad, Ali Moghimi, Mohammad Farhad Rahimi, Mohammad Reza Ghavamnasiri

**Affiliations:** 1 *Department of Biology, Faculty of Sciences, Ferdowsi University of Mashhad, Iran*; 2 *Department of Physics, Faculty of Sciences, Ferdowsi University of Mashhad, Iran*; 3 *Cancer Research Centre (CRC), Department of Radiation Oncology, Omid Hospital, Mashhad University of Medical Science, Mashhad, Iran*

**Keywords:** Gamma irradiation, Green tea, Lymphocytes, Micronucleus, Natural radioprotective

## Abstract

**Objective(s):**

The aim of this study was to investigate the radioprotective effect of green tea against genotoxicity induced by gamma irradiation in cultured blood lymphocytes from 5 human volunteers.

**Materials and Methods:**

Peripheral blood samples were collected from volunteers before and 1, 3 and 5 hr after drinking a decoction 4 g green tea in 280 ml boiling water for 5 constitutive days with the same quantity. At each time point, the whole blood samples were exposed to 200 cGy of ^60^ Co gamma irradiation and then were cultured with mitogenic stimulation to determine the chromosomal aberration in micronucleus assay on cytokinesis-blocked binucleated cells.

**Results:**

As expected, for each volunteer, the results showed a significant increase in the incidence of micronuclei after exposure to gamma irradiation as compared to non-irradiated control samples. Only lymphocytes blood sample collected 3 hr after drinking green tea exhibited a significant decrease in incidence of micronuclei compared to non-treated irradiated samples.

**Conclusion:**

These results suggest the radioprotective ability of green tea against ionizing radiation in human lymphocytes, at specified time after consumptior.

## Introduction

Intensional or unintentional irradiation due to radiotherapy during cancers or those caused by natural and industrial sources greatly damages the human tissues. Radiation exposure can induce damages either directly or indirectly to the genetic material of the cells. Indirect effects of radiation exposure are responsible for many biological damages to living tissues. The harmful effects of ionizing radiation are believed to be the result of interaction of free radicals with DNA or other cellular macromolecules (1, 2). Ionizing radiation generates free radicals while passing through living tissues. Interactions between free radicals and DNA can induce DNA damages and can lead to mutations and possibly cancer (3). With respect to side effects induced by ionizing radiation in patients undergoing radiotherapy or people exposed to it at their work place, the radioprotectors have an important role for tolerance and/or increasing survival rate in such people. Today, development of effective radioprotectives or modifiers is fast growing and is very important (4). Most of available radioprotectors are toxic at high doses or at recurrent usages and are expensive. One of the main radioprotective classes is thiol-based synthetic compounds such as amifostine. Amifostine is a powerful radioprotective compared to other agents (5), but has limited usage in clinical setting due to its side effects and toxicity (6).

The search for less toxic radiation protectors has spurred interest in the development of natural products. Natural products are suitable candidates for preventing harmful effects of ionizing irradiation, since they are non-toxic and have some proven therapeutic benefits (7). Natural products, such as herbal medicines, have only recently begun to receive some attention as possible modifiers of the radiation response (8, 9). Herbal extracts containing high amounts of flavonoids are a most effective of all (10, 11). Flavonoids are a family of polyphenolic compounds found in fruits and vegetables. They have wide range of biological properties including antibacterial, antiviral, anticancer, immunostimulant and antioxidant effects (12). Herbal plants have high levels of phenolic compounds that have radioprotective effects (4). One of the plants containing polyphenols is tea (13). Tea is a popular beverage worldwide. Tea, a product made up from leaf and bud of the plant* Camellia sinensis*, is the second most consumed beverage all over the world, well ahead of coffee and carbonated soft drinks. Among different teas, green tea has been chemically characterized; the water extract of green tea contains many polyphenols known as catechins that are known as antioxidants (14).

There is little information in the literature related to the radioprotective effects of green tea against ionizing radiation-induced chromosomal abnormalities in human lymphocytes. Therefore, the goal of this study was to determine the effect of green tea dinking on radiation-induced genetic damages in lymphocytes isolated from human volunteers. The damages induced by irradiation were investigated by the reliable method of micronucleus assay in cytokinesis-blocked binucleated lymphocytes. The use of binucleated cells in micronucleus assay was introduced in 1985 and developed later (15, 16). In this assay, the cytochinesis is blocked and any numerical or structural damages to chromosomes will be viewed as minute nuclei in binucleated cells called micronucleus (MnBi). Micronuclei may originate from either acentric chromosomal fragments or whole chromosome delayed in anaphase (15). This assay has been used widely to investigate the effects of different probable radioprotectors (8, 9).

## Materials and Methods

Informed consents were obtained from 5 healthy, non-smoking male human volunteers, aged between 20 and 25 years. The volunteers had almost the same low antioxidant diets during the experiment. Four g of commercially available green tea (Ahmad Tea) in 280 ml boiling water were steepened and drunk by each volunteer. Blood samples were collected in heparinized tubes from volunteers 1, 3 and 5 hr after drinking and at 1 hr after a daily doze of green tea for 5 constitutive days. At each sampling time, for each volunteer, 1 ml aliquots of heparinized whole blood was divided into two 25 ml culture flasks. One flask was the non-irradiated control sample and another was irradiated at 37 ^°^C with ^60^Co source (Theratron 780, Canada) with a dose of 200 cGy at a dose rate of 99.95 cGy/min. Subsequently, each sample (non-irradiated control and irradiated) was added to RPMI 1640 culture medium (Euroclone) supplemented by 20% fetal bovine serum (Gibco), 2% phytohaemagglutinin (Gibco), 100 µl/ml penicillin-streptomycin. Also, two cultures were set up, from each volunteer before drinking green tea, one left as non irradiated control and one was irradiated with the same dose of 99.95 cGy/min. All cultures were set up in duplicate and incubated at 37 ^°^C in a humidified atmosphere of 5% CO_2_/95% air. Cytochalasin B (Sigma-Aldrich, final concentration: 6 µg/ml) was added at 44 hr after culture initiation. At the end of 72 hr of incubation, the cells were collected according to Fenech (16) with small modification. Centrifuged cells were re-suspended in 0.056 g/l cold potassium chloride for 8 min and were fixed immediately in fixative solution (methanol: acetic acid, 6:1) three times. Fixed cells were dropped onto clean microscopic slides, air-dried and stained with 5% Giemsa. At least two slides per person were scored. All slides were coded by a separate scorer and were evaluated at ×1000 magnification for the frequency of micronuclei in cytokinesis-blocked binucleated cells with well preserved cytoplasm. Small nuclei were scored as micronuclei if their diameters were between 1/16 and 1/3 of main nuclei and were non-refractile, not linked to main nuclei and not overlapping the main nuclei (16). At each slide, a total number of 1000 binucleated cells were examined to record the micronucleus frequency.

**Figure 1:**
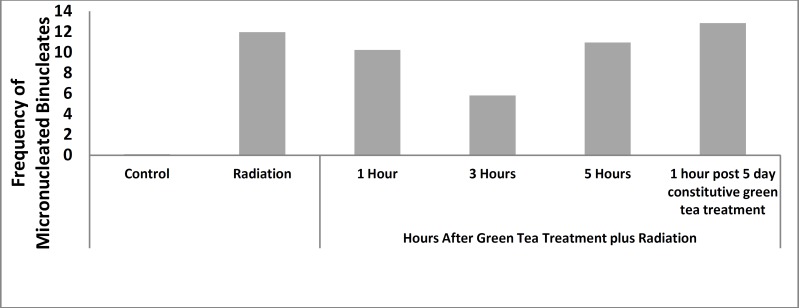
Frequency of micronucleated binucleates in green tea treated and non-treated volunteers after gamma-irradiation

The statistical analysis was performed by the software MINITAB. And the data was presented as mean.

## Results

The base line frequency of MnBi in non-irradiated blood lymphocytes from volunteers after drinking green tea at different time intervals before sampling were 0.06%±0.05 (Table 1). This frequency did not show any statistical differences with control (*P*< 0.01). This suggests that drinking green tea does not induce any chromosomal damages. The results show the statistically significant increase in the frequency of micronucleated binucleates (MnBi) after irradiation in all samples (11.96±2.10) compared to non-irradiated control cells (*P*< 0.001). The percentage of micronuclei induced by gamma-irradiation in lymphocytes of all volunteers at 1, 3 and 5 hr after drinking of green tea was 10.24±2.86, 5.82±1.36 and 10.69±0.9, respectively. The frequency of Mn in binucleated lymphocytes of volunteers who has drunk green tea for five constitutive dayswas 12.84±1.57.

**Table 1:** Effect of green tea on the frequency of micronucleated binucleates (MnBi) after gamma-irradiation

					Time interval between green tea treatment and irradiation					
		Control	Green tea	Radiation	1 hr	3 hr	5 hr	1 hr post 5 day constitutive green tea treatment		
Frequency of MnBi in five volunteers	1	0.2	0	15.5	14.9	7.5	9.8		13	
	2	0.1	0.1	12	8	7.1	11		13.7	
	3	0	0	11.4	10.8	4.8	10.4		11.1	
	4	0.1	0.1	10.8	8	5	12.1		11.5	
	5	0.1	0.1	10.1	9.5	4.7	11.5		14.9	
Mean±SD of MnBi%		0.1±0.07	0.06±0.05	11.96±2.10^a^	10.24±2.86^a^	5.82±1.36^a,b^	10.96±0.9^a^		12.84±1.57^a^	

^a^ *P*< 0.001 with control, ^b^ *P*< 0.001 with Radiation treated groups

**Figure 2:**
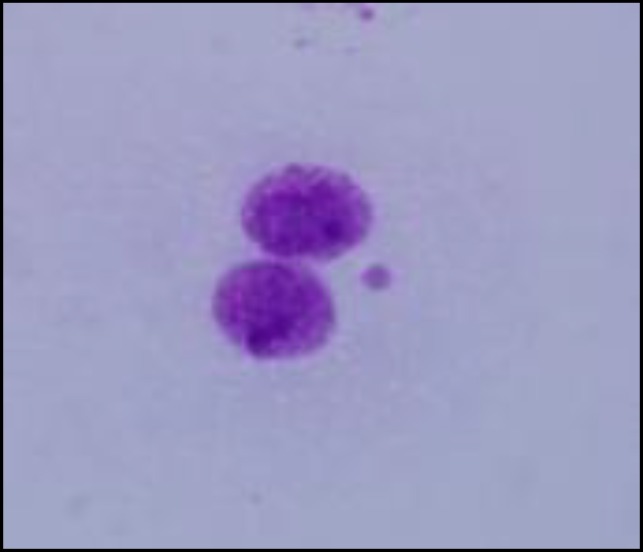
A typical micronucleated binucleates in this study

At all treatment schedules the frequency of micronucleated binucleates were statistically higher than in control group (*P*< 0.001). The result of green tea drinking at 1 and 5 hr before irradiation in both treatment schedules had no significant differences in their micronuclei with the non-treated irradiated lymphocytes. However, the lymphocytes in the blood samples were collected 3 hr after green tea drinking and were exposed *in vitro* to 200 cGy gamma-radiation. They exhibited a significant decrease in the incidence of micronuclei as compared to similarly non treated irradiated lymphocytes as well as other treated groups (*P*< 0.001). The total micronuclei values were 48.66% fold less 3 hr after green tea drinking (Table 1 and Figure 1). These data showed that green tea was almost rapidly absorbed and distributed through blood circulation after oral administration. A typical image of a binucleated cell with a micronucleus is shown in Figure 2.

## Discussion

Although many herbal preparations and compounds of plant origin have been evaluated as radioprotective agents in animals, there are few examples of experiments to demonstrate the efficacy of these extracts in human volunteers. A good radioprotective agent must provide different aspects for clinical applications, including effectiveness, low toxicity, availability, specificity and tolerance (17). Green tea, as one of the most widely used beverage all over the world, might fulfill these criteria. Green tea has been studied closely for its wide range of beneficial effects (14, 18, 19). Green tea is made up of polyphenols, mainly flavonoids and phenolic acids. Green tea polyphenols, also called catechins, are: (-)-epicatechin (EC), (-)-epicatechin gallate (ECG), (-)-epigallocatechin (EGC), and (-)-epigallocatechin-3-gallate (EGCG) (20). The antioxidant activity of green tea has been suggested in several studies (21, 22). This activity is related to its catechins. Protective effect of EGCG on neural cell death caused by post injury free radical induction in rats has been investigated (23). The antioxidant capability of catechins is related to its ability in scavenging the free radicals (24).

Regarding all the evidences about the antioxidant effect of green tea, the question remained unanswered is that whether drinking the green tea is able to protect the human cells from ionizing irradiation? In this study radioprotective effect of green tea was investigated.

Drinking of the green tea did not cause any chromosomal damages in human volunteers. However, it clearly reduced the frequency of chromosomal damages induced by gamma-irradiation. In an *in vitro* study on human lymphocytes with the same method of cytokinesis-blocked micronucleus assay, water soluble extract of green tea significantly reduced the X-ray induced Mn formation (25). Chromosomal damages induced by gamma-irradiation are mainly due to indirect effect of irradiation. Ionizing-irradiation turns on a long chain of events which leads to free radical formation (2). The protective effect of drinking green tea was related to its catechins. The radioprotectivity of flavonoids has been suggested in other studies (10, 11). Catechins in green tea act effectively as a free radical scavenger to reduce the damages caused by ionizing-induced free radicals. Green tea polyphenols showed to be able to reduce the harmful effect of whole body irradiation in mice. From all green tea polyphenol the catechins revealed to be most effective (26). 

Short or long term drinking of green tea did not show any differences in its capability in reducing the chromosomal damages induced by gamma-irradiation in this study. There was a cell protection at only 3 hr post drinking. The peak plasma concentration of metabolite conjugates of catechins was reported to be around two hr post drinking (27, 28). Therefore, we should expect the highest protection of the blood lymphocytes to be at the same time of the peak of the metabolite conjugates of catechin.

The results here suggest that green tea could be a useful candidate for protection of occupationally exposed individuals, such as those working in medical practice involving radiotherapy. For better protection one should drink green tea at around 2-3 hr before possible exposure. 

## Conclusions

The data suggest that green tea could be a potential radioprotector when consumed 3 hr before irradiation. It could clearly reduce the gamma-induced chromosomal damages. This reduction in the frequency of micronuclei was not observed in other treatments.
